# Early estimation of the risk factors for hospitalization and mortality by COVID-19 in Mexico

**DOI:** 10.1371/journal.pone.0238905

**Published:** 2020-09-11

**Authors:** María Fernanda Carrillo-Vega, Guillermo Salinas-Escudero, Carmen García-Peña, Luis Miguel Gutiérrez-Robledo, Lorena Parra-Rodríguez

**Affiliations:** 1 Research Department, Instituto Nacional de Geriatría, Mexico City, Mexico; 2 Center for Economic and Social Studies in Health, Hospital Infantil de México Federico Gómez, Mexico City, Mexico; Azienda Ospedaliero Universitaria Careggi, ITALY

## Abstract

**Background:**

Due to a high prevalence of chronic non-degenerative diseases, it is suspected that COVID 19 poses a high risk of fatal complications for the Mexican population. The present study aims to estimate the risk factors for hospitalization and death in the Mexican population infected by SARS-CoV-2.

**Methods and findings:**

We used the publicly available data released by the Epidemiological Surveillance System for Viral Respiratory Diseases of the Mexican Ministry of Health (Secretaría de Salud, SSA). All records of positive SARS-CoV-2 cases were included. Two multiple logistic regression models were fitted to estimate the association between hospitalization and mortality, with other covariables. Data on 10,544 individuals (57.68% men), with mean age 46.47±15.62, were analyzed. Men were about 1.54 times more likely to be hospitalized than women (p<0.001, 95% C.I. 1.37–1.74); individuals aged 50–74 and ≥74 were more likely to be hospitalized than people aged 25–49 (OR 2.05, p<0.001, 95% C.I. 1.81–2.32, and OR 3.84, p<0.001, 95% C.I. 2.90–5.15, respectively). People with hypertension, obesity, and diabetes were more likely to be hospitalized than people without these comorbidities (p<0.01). Men had more risk of death in comparison to women (OR = 1.53, p<0.001, 95% C.I. 1.30–1.81) and individuals aged 50–74 and ≥75 were more likely to die than people aged 25–49 (OR 1.96, p<0.001, 95% C.I. 1.63–2.34, and OR 3.74, p<0.001, 95% C.I. 2.80–4.98, respectively). Hypertension, obesity, and diabetes presented in combination conveyed a higher risk of dying in comparison to not having these diseases (OR = 2.10; p<0.001, 95% C.I. 1.50–2.93). Hospitalization, intubation and pneumonia entail a higher risk of dying (OR 5.02, p<0.001, 95% C.I. 3.88–6.50; OR 4.27, p<0.001, 95% C.I. 3.26–5.59, and OR = 2.57; p<0.001, 95% C.I. 2.11–3.13, respectively). Our study’s main limitation is the lack of information on mild (asymptomatic) or moderate cases of COVID-19.

**Conclusions:**

The present study points out that in Mexico, where an important proportion of the population has two or more chronic conditions simultaneously, a high mortality rate is a serious risk for those infected by SARS-CoV-2.

## Introduction

Since the start of the COVID-19 epidemic in Wuhan, China, in December 2019, SARS-CoV-2 has continued to spread globally, resulting in more than 3 million confirmed cases and about 230 thousand deaths worldwide by the first week of May 2020 [1]. The up-to-date data evidence that the growth of the pandemic had slowed in Asia, while Europe and America contribute the highest number of cases worldwide. The latter is the region with the highest incidence, contributing 49% of cases across the World Health Organization (WHO) regions in the last 14 days of April. Since the first case reported in America by The United States on January 31^st^, SARS-CoV-2 has spread to 50 countries and territories in the region, including Mexico [2]. This country confirmed the first case of COVID-19 on February 27^th^ and the first death on March 19^th^. On March 23^rd^, Mexican health authorities declared COVID-19 a health emergency, instating social isolation as the main action to contain the epidemic [3]. The acceleration phase in Mexico was declared almost a month later, on April 21^st^, when the number of confirmed cases was 9,501 and 857 total deaths had been registered.

The first information on COVID-19 was generated in China, where the disease transmission mechanisms, the incubation period, and the clinical manifestations had been described. [4–7]. It has been stated that most of the infected individuals recover spontaneously in about 7 to 10 days. While the rest develop fatal complications including organ failure, septic shock, pulmonary edema, severe pneumonia, and Acute Respiratory Distress Syndrome (ARDS) [5]. Older people and those with underlying health conditions are at higher risk of severe disease and death [8].

Although the aging rate is not as high as in other countries, Mexico has one of the highest prevalences of chronic non-degenerative diseases; especially obesity, diabetes, and hypertension, in both young and older adults [9]. Consequently, it is suspected that the risk of fatal complications due to COVID-19 is higher than in other countries.

Amidst the global health emergency and considering that the knowledge of such information is crucial for the government and health authorities in the decision-making process during the epidemic, this work aims to estimate the risk factors for hospitalization and death in the Mexican population infected by SARS-CoV-2.

## Methods

The present analysis was based on the publicly available data of the suspected cases of viral respiratory disease released by the Epidemiological Surveillance System for Viral Respiratory Diseases of the Mexican Ministry of Health (Secretaría de Salud, SSA) on April 23rd, 2020 [10]. This system contains data collected by 475 viral respiratory disease monitoring units (USMER) located in the different health services throughout the country, and directly from the medical units that attended the cases.

The dataset includes positive, negative, and suspected cases of COVID, with or without pneumonia, both in ambulatory and hospital management. Information on sex, age, nationality, place of residence, and migratory status was registered. The information includes the type of medical unit of first contact (USMER or not USMER), whether the individual was hospitalized or stayed in outpatient management, and if the service was public or private. The dates when the individual developed COVID-19 symptoms, when the person was admitted to hospitalization, and the date of death were recorded. Comorbidities encompassed hypertension, diabetes, obesity, cardiovascular disease, chronic obstructive pulmonary disease (COPD), asthma, chronic kidney disease (CKD), immunosuppression, and other diseases reported by the individual. Other risk factors for severe COVID-19 included smoking and pregnancy. The database does not contain the evolution of the patient during the stay in the medical units.

For the present analysis, all records of positive SARS-CoV-2 cases were included. No record was deleted due to the presence of missing data since the number of missing values was <2% of the total registers. The data cut-off for the study was April 23rd, 2020.

### Variables

Two primary endpoints were defined: hospitalization, and death. The former defined as individuals who tested positive for COVID-19 and required hospitalization. The latter was defined as positive cases for COVID-19 who died, regardless of being hospitalized or remaining in outpatient management.

Variables about multimorbidity and risk factors were collected directly from the dataset, specifications about definitions are not included.

Age was used as a continuous variable for describing the sample and as a categorical variable with four groups (<25 years old, 25–49 years old, 50–74 years old, and ≥75 years old) for the rest of the analysis. Sex was used as a dichotomic variable (man or woman).

Hypertension, obesity, diabetes, cardiovascular disease, chronic kidney disease (CKD), chronic obstructive pulmonary disease (COPD), pregnancy, immune-suppression condition, smoking, development of pneumonia, intubation, and admission to ICU were analyzed as dichotomic variables (“yes” or “no”). Days from the presentation of symptoms to hospitalization and death, and from admission to a health care unit to death, were analyzed as continuous variables.

Fourteen options for the health service that provided the treatment were available in the dataset. From these, five variables were created with two possible categories (“yes” or “no”): IMSS (Mexican Institute of Social Security), ISSSTE (Institute of Social Security and Services for State Workers), Private services, SSA (Health Ministry) and other services, that includes the Red Cross, DIF (National System for the Integral Development of the Family), IMSS-BIENESTAR, the municipal health services, PEMEX (Health services of Petróleos Mexicanos), health services of the armed forces (SEDENA and SEMAR) and university hospitals. For the regression models, the health services variable groups these options into five categories.

Because of the high prevalence of chronic diseases in Mexico, especially hypertension, diabetes, and obesity, in addition to reporting its prevalence alone, these morbidities were grouped to determine if their combined effects involve a different risk. The combinations were categorized as “present” or “not present”.

### Statistical analysis

A descriptive analysis was performed. Continuous variables are presented as means and standard deviations, categorical variables are expressed as number and percentage. Comparisons of hospitalized *v* not hospitalized individuals, and survivors *v* those who did not survive were estimated through the Mann-Whitney test for continuous variables and χ^2^ for categorical variables. From this point, only individuals over 25 years old were included in the analysis, as in those 25 and younger, only 5 deaths had been registered.

Two multiple logistic regression models were fitted to estimate the association between hospitalization and mortality, with the rest of the covariables. Those variables that resulted not significant were excluded from the final model. All analyses were performed with the statistical package software STATA 14.

## Results

Data on 10,544 individuals with mean age 46.47±15.62 were analyzed. From this total, 57.68% (n = 6,082) were men (Table 1). From the general population, 55.61% (n = 5797) had no comorbidities. The prevalence and number of morbidities increased as age did. Hypertension was the most prevalent morbidity (21.74%), followed by obesity (20.05%) and diabetes (17.65%).

**Table 1:** General characteristics by age group.

	Total	<25 y	25–49 y	50–74 y	≥75 y
	n = 10544	n = 598	n = 5640	n = 3823	n = 483
**Sex (men)**	6082 (57.68)	329 (55.02)	3203 (56.79)	2286 (59.8)	264 (54.66)
**Hypertension**	2272 (21.74)	7 (1.17)	622 (11.11)	1372 (36.31)	271 (56.58)
**Obesity**	2097 (20.05)	44 (7.37)	1115 (19.91)	864 (22.84)	74 (15.48)
**Diabetes**	1845 (17.65)	11 (1.84)	525 (9.38)	1148 (30.38)	161 (33.68)
**Cardiovascular Disease**	312 (2.99)	2 (0.34)	57 (1.02)	197 (5.22)	56 (11.69)
**Chronic Kidney Disease**	222 (2.13)	3 (0.5)	61 (1.09)	135 (3.58)	23 (4.81)
**COPD**	271 (2.59)	1 (0.17)	39 (0.7)	156 (4.13)	75 (15.66)
**Asthma**	374 (3.58)	18 (3.02)	243 (4.34)	101 (2.68)	12 (2.51)
**Number of Comorbidities**	0.7 ± 0.96	0.14 ± 0.42	0.47 ± 0.77	1.04 ± 1.08	1.4 ± 1.15
**No Comorbidities**	5797 (55.61)	524 (88.07)	3692 (66.05)	1465 (38.91)	116 (24.42)
**Combined Chronic Diseases**					
** Diabetes & Hypertension & Obesity**	323 (3.06)	1 (0.17)	86 (1.52)	207 (5.41)	29 (6.0)
** Diabetes & Hypertension**	638 (6.05)	0	115 (2.04)	440 (11.51)	83 (17.18)
** Diabetes & Obesity**	232 (2.2)	4 (0.67)	109 (1.93)	115 (3.01)	4 (0.83)
** Hypertension & Obesity**	395 (3.75)	0	163 (2.89)	207 (5.41)	25 (5.18)
** Only Hypertension**	914 (8.67)	6 (1.0)	258 (4.57)	517 (13.52)	133 (27.54)
** Only Obesity**	1142 (10.83)	39 (6.52)	756 (13.4)	331 (8.66)	16 (3.31)
** Only Diabetes**	649 (6.16)	6 (1.0)	215 (3.81)	384 (10.04)	44 (9.11)
**Pregnancy**	54 (0.51)	6 (1.01)	48 (0.85)	--	--
**Immuno-Suppressed**	207 (1.98)	12 (2.01)	76 (1.36)	99 (2.62)	20 (4.18)
**Smoking**	936 (8.96)	42 (7.04)	517 (9.24)	322 (8.53)	55 (11.48)
**Pneumonia**	3024 (28.68)	53 (8.86)	1122 (19.89)	1581 (41.35)	268 (55.49)
**Hospitalized**	3997 (37.91)	75 (12.54)	1532 (27.16)	2040 (53.36)	350 (72.46)
**Intubated**	466 (4.42)	6 (1.01)	123 (2.18)	289 (7.56)	48 (9.94)
**ICU**	492 (4.67)	6 (1.01)	153 (2.71)	296 (7.74)	37 (7.66)
**Dead**	968 (9.18)	5 (0.84)	255 (4.52)	573 (14.99)	135 (27.95)
**Days Symptoms-Admission**	4.32 ± 3.39	3.86 ± 3.11	4.26 ± 3.26	4.46 ± 3.56	4.56 ± 3.74
**Days Admission-Death**	5.88 ± 4.95	2 ± 2.34	6.12 ± 4.61	5.83 ± 5.03	5.82 ± 5.24
**Days Symptoms-Death**	10.08 ± 5.47	7 ± 3.39	10.23 ± 4.95	9.95 ± 5.71	10.47 ± 5.41
**IMSS Services**	3634 (34.47)	115 (19.23)	2185 (38.74)	1189 (31.1)	145 (30.02)
**ISSSTE Services**	641 (6.08)	12 (2.01)	274 (4.86)	293 (7.66)	62 (12.84)
**SSA Services**	5108 (48.44)	398 (66.56)	2631 (46.65)	1878 (49.12)	201 (41.61)
**Other Public Services**	415 (3.94)	12 (2.01)	175 (3.1)	190 (4.97)	38 (7.87)
**Private Services**	746 (7.08)	61 (10.2)	375 (6.65)	273 (7.14)	37 (7.66)

When considering the simultaneous presence of chronic diseases, the pair diabetes-hypertension was the most frequent (6.05%), followed by hypertension-obesity (3.75%), and diabetes-obesity (2.2%). Table 1 shows that 323 individuals (3.06%) present diabetes, hypertension, and obesity simultaneously. Obesity in combination with hypertension (5.41%) and diabetes (3.01%) was more frequent in individuals from 50–74 years old. Obesity alone was more prevalent (13.4%) in the 25–49 age group.

Of the total number of COVID-19 cases, 28.68% developed pneumonia, this was more frequent in individuals over 50 (61.14% of the total cases), and 37.91% were hospitalized, mostly individuals over 50 (59.79%). Hospitalization, intubation, and intensive care unit admission were more frequent in older age groups.

From the total individuals, 968 (9.18%) died. Deaths increased as age did, with a higher percentage in older age groups. Death occurred in 9.18% of the total number of cases, 0.84% in individuals under 25, 4.52% in the 25–49 group, 14.99% in people between 50 and 74, and 27.95% for the oldest age group. The mean of days from the presentation of symptoms to the admission was 4.32±3.39 days; from the admission to death 5.88±4.95 days elapsed, and from the presentation of symptoms to death passed 10.08±5.47 days. The health ministry services (SSA, 48.44%), and the Mexican Institute of Social Services (IMSS, 34.47%) were the institutions that treated the largest number of cases (Table 1).

Table 2 presents the results of the comparisons between the two main outcomes. Most hospitalized individuals were men; people in this group were older, with a higher prevalence of comorbidity, both alone and combined (p≤0.01). Immuno-suppression and smoking were also more frequent in the patients who were hospitalized; pneumonia, intubation, and admission to the ICU were especially higher in this group (p≤0.01). The mortality rate was higher for men than it was for women. People who died were older, with a higher prevalence of single and combined comorbidities(p≤0.01) than survivors. Hospitalization, ICU admission, and intubation were more frequent among patients who did not survive (p≤0.01). A higher percentage of those who did not survive were treated at IMSS, while survivors were more frequently treated by SSA services (p≤0.01). For both outcomes, obesity alone, the days between the presentation of symptoms, admission to hospital, and death, were no different (p≥0.05).

**Table 2:** General characteristics by outcome.

	Total	Not Hospitalized	Hospitalized	p value	Survivor	Not a Survivor	p value
	n = 9946	n = 6024	n = 3922		n = 8983	n = 963	
**Age, years**	48.15 ± 14.35	44.18 ± 12.9	54.24 ± 14.34	<0.0001	46.96 ± 13.91	59.26 ± 13.63	0.0001
**Sex (men)**	5753 (57.84)	3204 (53.19)	2549 (64.99)	0.0001	5086 (56.62)	667 (69.26)	0.0001
**Hypertension**	2265 (22.98)	938 (15.73)	1327 (34.1)	0.0001	1845 (20.74)	420 (43.8)	0.0001
**Obesity**	2053 (20.82)	1066 (17.86)	987 (25.35)	0.0001	1764 (19.82)	289 (30.1)	0.0001
**Diabetes**	1834 (18.61)	668 (11.2)	1166 (29.97)	0.0001	1470 (16.53)	364 (37.92)	0.0001
**Cardiovascular Disease**	310 (3.15)	110 (1.84)	200 (5.14)	0.0001	246 (2.77)	64 (6.68)	0.0001
**Chronic Kidney Disease**	219 (2.22)	54 (0.91)	165 (4.24)	0.0001	156 (1.75)	63 (6.58)	0.0001
**COPD**	270 (2.74)	72 (1.21)	198 (5.09)	0.0001	187 (2.1)	83 (8.65)	0.0001
**Asthma**	356 (3.61)	237 (3.98)	119 (3.06)	0.0177	325 (3.66)	31 (3.23)	0.4925
**Number of Comorbidities**	0.74 ± 0.97	0.52 ± 0.82	1.07 ± 1.08	<0.0001	0.67 ± 0.92	1.36 ± 1.16	<0.0001
**No Comorbidities**	5273 (53.64)	3812 (64.01)	1461 (37.7)	0.0001	5018 (56.54)	255 (26.7)	0.0001
**Combined Chronic Diseases**							
** Diabetes & Hypertension & Obesity**	322 (3.24)	122 (2.03)	200 (5.1)	0.0001	246 (2.74)	76 (7.89)	0.0001
** Diabetes & Hypertension**	638 (6.41)	193 (3.2)	445 (11.35)	0.0001	485 (5.4)	153 (15.89)	0.0001
** Diabetes & Obesity**	228 (2.29)	100 (1.66)	128 (3.26)	0.0001	190 (2.12)	38 (3.95)	0.0003
** Hypertension & Obesity**	395 (3.97)	192 (3.19)	203 (5.18)	0.0001	333 (3.71)	62 (6.44)	0.0001
** Only Hypertension**	908 (9.13)	431 (7.15)	477 (12.16)	0.0001	779 (8.67)	129 (13.4)	0.0001
** Only Obesity**	1103 (11.09)	650 (10.79)	453 (11.55)	0.2385	990 (11.02)	113 (11.73)	0.5028
** Only Diabetes**	643 (6.46)	253 (4.2)	390 (9.94)	0.0001	547 (6.09)	96 (9.97)	0.0001
**Pregnancy**	48 (0.48)	34 (0.56)	14 (0.36)	0.1525	43 (0.48)	5 (0.52)	0.7329
**Immuno-Suppressed**	195 (1.98)	79 (1.32)	116 (2.98)	0.0001	149 (1.68)	46 (4.8)	0.0001
**Smoking**	894 (9.07)	486 (8.15)	408 (10.49)	0.0001	795 (8.94)	99 (10.33)	0.1524
**Pneumonia**	2971 (29.87)	327 (5.43)	2644 (67.41)	0.0001	2245 (24.99)	726 (75.39)	0.0001
**Hospitalized**	3922 (39.43)	--	3922 (100)	0.0001	3054 (34).0	868 (90.13)	0.0001
**Intubated**	460 (4.63)	--	460 (11.73)	0.0001	234 (2.61)	226 (23.47)	0.0001
**ICU**	486 (4.89)	--	486 (12.39)	0.0001	294 (3.27)	192 (19.94)	0.0001
**Dead**	963 (9.68)	95 (1.58)	868 (22.13)	0.0001	--	963 (100)	0.0001
**Days Symptoms-Admission**	4.35 ± 3.4	4.31 ± 3.3	4.41 ± 3.55	0.1631	4.37 ± 3.38	4.19 ± 3.62	0.1107
**Days Admission-Death**	5.9 ± 4.95	6.03 ± 5.33	5.89 ± 4.91	0.8005	--	5.93 ± 4.93	0.8005
**Days Symptoms-Death**	10.09 ± 5.47	10.79 ± 6.38	10.02 ± 5.36	0.1920	--	10.12 ± 5.45	0.1920
**IMSS Services**	3519 (35.38)	2023 (33.58)	1496 (38.14)	0.0001	3069 (34.16)	450 (46.73)	0.0001
**ISSSTE Services**	629 (6.32)	212 (3.52)	417 (10.63)	0.0001	562 (6.26)	67 (6.96)	0.3945
**SSA Services**	4710 (47.36)	3137 (52.08)	1573 (40.11)	0.0001	4331 (48.21)	379 (39.36)	0.0001
**Other Public Services**	403 (4.05)	191 (3.17)	212 (5.41)	0.0001	359 (4)	44 (4.57)	0.3874
**Private Services**	685 (6.89)	461 (7.65)	224 (5.71)	0.0002	662 (7.37)	23 (2.39)	0.0001

Although pregnant women represent a small subgroup (n = 48), the frequency of diabetes and obesity among those who died (n = 5) is striking. Diabetes alone was present in 40% and combined with obesity in 20% of the fatal cases, while in those who survived, no cases were observed (p = 0.000 for diabetes and p = 0.0034 for diabetes and obesity). Obesity was present in 20% of the women who did not survive and in 9.3% of the survivors (p = 0.4633). Only 20% of the fatal cases did not have these diseases compared to 86.05% of no disease in those who survived (p = 0.0007).

The full logistic regression model for hospitalization containing seven covariates (Table 3) was statistically significant (n = 9847; Goodness-of-fit test x^2^ = 1080.18, df = 536, p<0.001; Area under the ROC curve = 0.8886). Controlling for other predictors in the model, men were about 1.54 times more likely to be hospitalized than women (p<0.001, 95% C.I. 1.37–1.74); individuals aged 50–74 and ≥74 were more likely to be hospitalized than people aged 25–49 (OR 2.05, p<0.001, 95% C.I. 1.81–2.32, and OR 3.84, p<0.001, 95% C.I. 2.90–5.15, respectively). People with chronic kidney disease and with COPD was 2.01 and 1.73 times more likely to be hospitalized than people without these comorbidities (p = 0.001, 95% C.I. 1.33–3.04, and p = 0.003, 95% C.I. 1.20–2.50, respectively).

**Table 3:** Logistic regression model for the risk of hospitalization.

	Odds Ratio	Std. Err.	z	p>z	95% C.I.	
**Men**	1.54	0.09	7.32	<0.001	1.37	1.74
**Age 25–49 years (reference)**						
** Age 50–74 years**	2.05	0.13	11.31	<0.001	1.81	2.32
** Age ≥ 75 years**	3.84	0.56	9.32	<0.001	2.90	5.10
**CKD**	2.01	0.42	3.33	0.001	1.33	3.04
**COPD**	1.73	0.32	2.96	0.003	1.20	2.50
**None (reference)**						
** Diabetes & Hypertension & Obesity**	1.85	0.31	3.74	<0.001	1.34	2.56
** Diabetes & Hypertension**	2.60	0.32	7.73	<0.001	2.04	3.31
** Diabetes & Obesity**	1.78	0.34	3.01	0.003	1.22	2.59
** Hypertension & Obesity**	1.65	0.24	3.45	0.001	1.24	2.19
** Only Hypertension**	1.54	0.16	4.2	<0.001	1.26	1.88
** Only Obesity**	1.64	0.15	5.41	<0.001	1.37	1.95
** Only Diabetes**	2.14	0.25	6.43	<0.001	1.70	2.69
**Pneumonia**	33.62	2.40	49.14	<0.001	29.22	38.68
**Private Services (reference)**						
** IMSS Services**	2.15	0.28	5.85	<0.001	1.67	2.78
** ISSSTE Services**	3.08	0.52	6.68	<0.001	2.21	4.28
** SSA Services**	0.82	0.11	-1.56	0.118	0.63	1.05
** Other Services**	1.72	0.33	2.88	0.004	1.19	2.49
**Constant**	0.03	0.01	-14.61	<0.001	0.02	0.04

The risk of hospitalization for all the possible combinations of the presence of diabetes, hypertension, and obesity was statistically significant when compared with not having any of the three main chronic diseases. The combination diabetes-hypertension had the highest risk of hospitalization (OR = 2.60, 95% C.I.2.04–3.31), followed in second place by diabetes alone (OR = 2.14, 95% C.I.1.70–2.69), and in third place by having diabetes, hypertension, and obesity simultaneously (OR = 1.85, 95% C.I.1.34–2.56). People with hypertension alone presented the smallest risk of being hospitalized (OR = 1.54, 95% C.I. 1.26–1.88) except for individuals without the three main comorbidities.

People treated at ISSSTE, IMSS, and other public services were more likely to be hospitalized than people treated at private services (OR = 3.08, 2.15, and 1.72, respectively). Developing pneumonia resulted in the highest OR to be hospitalized (OR = 33.62, p<0.001, 95% C.I. 29.22–38.68) as opposed to not having pneumonia.

The full logistic regression model for mortality containing twelve covariates (Table 4) was statistically significant (n = 9845; Goodness-of-fit test x^2^ = 2180.73, df = 1140, p<0.001; Area under the ROC curve = 0.8840). Controlling for other predictors in the model, the highest risk of death was given by IMSS in comparison with private services (OR 9.25, p<0.001, 95% C.I. 5.61–15.24); for the same variable, SSA, other and ISSSTE services had more risk of death in comparison to private services (OR = 3.56, 2.45 and 2.25). Men had more risk of dying in comparison to women (OR = 1.53, p<0.001, 95% C.I. 1.30–1.81) and individuals aged 50–74 and ≥75 were more likely to die than people aged 25–49 (OR 1.96, p<0.001, 95% C.I. 1.63–2.34, and OR 3.74, p<0.001, 95% C.I. 2.80–4.98, respectively). People with chronic kidney disease and COPD had 1.44 and 1.68 times more risk of dying than people without these comorbidities (p = 0.047, 95% C.I. 1.01–2.06, and p = 0.002, 95% C.I. 1.22–2.31, respectively).

**Table 4:** Logistic regression model for the risk of death.

	Odds Ratio	Std. Err.	z	p>z	95% C.I.	
**Men**	1.53	0.13	5.01	<0.001	1.30	1.81
**Age 25–49 years (reference)**						
** Age 50–74 years**	1.96	0.18	7.29	<0.001	1.63	2.34
** Age ≥ 75 years**	3.74	0.55	9.01	<0.001	2.80	4.98
**CKD**	1.44	0.26	1.99	0.047	1.01	2.06
**COPD**	1.68	0.27	3.16	0.002	1.22	2.31
**None (reference)**						
** Diabetes & Hypertension & Obesity**	2.10	0.36	4.32	<0.001	1.50	2.93
** Diabetes & Hypertension**	1.92	0.25	4.94	<0.001	1.48	2.49
** Diabetes & Obesity**	2.06	0.44	3.39	0.001	1.35	3.12
** Hypertension & Obesity**	1.88	0.33	3.58	<0.001	1.33	2.65
** Hypertension**	1.49	0.19	3.04	0.002	1.15	1.92
** Obesity**	1.74	0.23	4.21	<0.001	1.35	2.26
** Diabetes**	1.50	0.21	2.81	0.005	1.13	1.98
**Pregnancy**	3.56	2.01	2.24	0.025	1.17	10.80
**Immuno-Suppressed**	1.70	0.35	2.57	0.010	1.13	2.55
**Pneumonia**	2.57	0.26	9.45	<0.001	2.11	3.13
**Hospitalized**	5.02	0.66	12.25	<0.001	3.88	6.50
**Intubated**	4.27	0.59	10.51	<0.001	3.26	5.59
**ICU**	1.79	0.25	4.18	<0.001	1.36	2.36
**Private Services (reference)**						
** IMSS Services**	9.25	2.36	8.72	<0.001	5.61	15.24
** ISSSTE Services**	2.25	0.64	2.86	0.004	1.29	3.91
** SSA Services**	3.56	0.89	5.08	<0.001	2.18	5.81
** Other Services**	2.45	0.75	2.93	0.003	1.34	4.46
**Constant**	0.00	0.00	17.18	<0.001	0.00	0.00

The risk of death for the seven possible combinations of the presence of diabetes, hypertension, and obesity, were statistically significant when compared with not having these diseases. Presenting the three comorbidities simultaneously had the highest risk of dying (OR = 2.10, 95% C.I. 1.50–2.93), followed by having the combination diabetes-obesity (OR = 2.06, 95% C.I. 1.35–3.12) and by the pair diabetes-hypertension (OR = 1.92, 95% C.I. 1.48–2.49). People with hypertension alone presented the smallest risk of dying (OR = 1.49, 95% C.I. 1.15–1.92) except for individuals without the three main comorbidities.

Pregnant women had 3.56 times the risk of dying compared with non-pregnant women (p = 0.025, 95% C.I. 1.17–10.80), and immuno-suppressed individuals are about 1.70 times more likely to die than people without this condition. People who developed pneumonia were 2.57 times more likely to die than people without it (p<0.001, 95% C.I. 2.11–3.13). Those who were hospitalized, intubated, and admitted to ICU had a higher risk of dying than their counterparts who were not (OR 5.02, p<0.001, 95% C.I. 3.88–6.50, OR 4.27, p<0.001, 95% C.I. 3.26–5.59, and OR 1.79, p<0.001, 95% C.I. 1.36–2.36, respectively).

## Discussion

To our knowledge, this is the first study of COVID-19 reporting the risk factors for hospitalization and death in the Mexican population. The likelihood to be hospitalized increased as a result of the following factors or any combination of them: being male; belonging to an older age group; having CKD, COPD, a chronic disease or a combination of them; developing pneumonia; or being treated at a public health institution. The same factors in addition to pregnancy, immuno-suppression, hospitalization, intubation, and admission to the ICU, increased the risk of death. The same factors, in addition to pregnancy, immuno-suppression, hospitalization, intubation, and admission to the ICU, increased the risk of death.

Shortly after COVID-19 had been declared a pandemic, small case series of individuals treated in different hospitals in China were reported [11–13]. Endpoints, especially admission to ICU and invasive ventilation, were frequently reported. Hospitalization, one of the events of significant concern to health systems due to the risk of saturation, was used as a primary outcome. In fact, a high hospitalization rate was reported by Guan et al. [14], who informed that 93.6% of the individuals with COVID-19 received hospital care. In-hospital mortality was reported to be as high as 28% and 97% for individuals requiring mechanical ventilation [15]. In the present analysis, 40% of the individuals were treated in hospitals. Of the 3922 hospitalized individuals, 67% developed pneumonia, 88% were intubated, and 12% were admitted to the ICU. The death rate within hospitalization was 22%.

Overall lethality of COVID-19 in the present study was 9.2%. The higher proportion of deaths was observed for the oldest age group (27.95%), and in individuals with any comorbidity (73.3%).

Li et al. published a meta-analysis that aimed to analyze the clinical data, discharge rate, and fatality rate of COVID-19 patients for clinical help. Male sex was strongly related to adverse outcomes [16]. In the present analysis, men also have more risk of hospitalization and death, 54% and 53%, respectively, than women. It has been repeatedly informed that fatality rates for males are two to three times higher than for females [17]. Wenham et al. [18] suggested that gender-related social factors, immunological differences, hormonal disparities, and lifestyle habits may play a role in the sex differences for COVID-19.

Regarding age, in Europe, a higher mortality rate has been reported in older age groups [19]. A similar pattern has been reported for China [20]. It could be thought that in Mexico the risk for the older population would be lower, as the elderly group is smaller compared to those countries. Even so, hospitalization and mortality risks were more than twice for the older age groups in our analysis. The reduced immune response and the increased prevalence of multimorbidity that characterized this age group can explain the higher risk of both outcomes.

Similarly, comorbidity increased the risk of hospitalization and death in the present analysis, mainly when chronic degenerative diseases co-occur. Of the individuals requiring hospitalization, 62% had comorbidities, primarily hypertension (34%), diabetes (30%), and obesity (25%). The presence of these three diseases in the same person increases the risk of hospitalization by 85%. For mortality, a similar pattern can be observed. In individuals who died, hypertension was present in 44%, diabetes in 38%, and obesity in 30%. The risk of dying in individuals presenting the combination of these three diseases was 2.10 times the risk compared with those without them. It is important to note that individuals who died with no comorbidities (27%) were younger (56.17±13.42 years) than those who died and did have morbidities (60.41±13.59 years). In Mexico, a high proportion of undiagnosed chronic diseases has been reported [21], it can be hypothesized that a percentage of hospitalizations and deaths from COVID-19 can be related to undiagnosed morbidities.

Data on pregnancy and COVID-19 is limited, but based on the experience with influenza, SARS, and MERS, pregnant women, especially those in the second and third trimesters of gestation, have a higher risk of complications and death in comparison with non-pregnant women [22–24]. In the present analysis, pregnant women, in comparison to non-pregnant, were 3.56 times more likely to die because of coronavirus. It must be noted that 80% of pregnant women who died, also had obesity, or diabetes.

Hospitalization in this population seems to act as a strong risk factor for dying, and this risk further increased when individuals were admitted to the ICU and were intubated. This association can be related to the fact that most people with COVID-19 who accessed health services are complicated cases of COVID-19.

Concerning health services, it was observed that the risk of dying is more than twice in public services than in private ones. Of especial interest is the fact that being treated at IMSS resulted in 8.25 times the risk of dying. Public hospitals in Mexico are the health services with the highest demand, as are more affordable and accessible to most of the Mexican population. These institutions are at risk of exceeding their response capacity, increasing the severity and death rates associated with health services saturation. The stark contrast of outcomes raises the issue of inequality that hinders access to quality care due to late arrivals, overcrowded services, and inadequate staffing in public hospital *v* world-class care in the private sector.

This study has some limitations. First, given the dynamics of the disease and that in Mexico there are insufficient resources to apply tests massively. Rates were calculated based on sentinel information that includes all deaths, but not all mild (asymptomatic) or moderate cases that did not resort to health services. As a result, the prevalence of the disease could be underestimated, whereas lethality could be overestimated. Therefore, when interpreting the data, it would be convenient to consider the estimation of real cases, which are mostly mild cases of the disease. Second, more detailed patient information, mainly, dates of hospital discharge of patients who do not die, was unavailable at the time of analysis; this information would be of vital importance to assess the possible saturation of health services and to assess the use of resources.

Nonetheless, this study is, to our knowledge, the largest case series to date of COVID-19 in Mexico, with 10,544 individuals from all over the country, and provides further information on patients’ clinical and epidemiological features. It presents the latest status of COVID-19 in Mexico, and a wide range of clinical manifestations can be seen and are associated with adverse outcomes.

## Conclusions

COVID-19 places a substantial strain on health systems worldwide. For countries starting the accelerated contagion phase, it is crucial to identify poor prognoses at an early stage to allocate limited resources better. In this respect, the present study points out that in Mexico, where a vast proportion of the population develops two or more chronic conditions simultaneously, a high mortality rate is a serious risk for those infected by SARS-CoV-2.

## Supporting information

S1 Material (CSV)
